# A cluster randomized controlled trial of an after-school playground curriculum intervention to improve children’s physical, social, and emotional health: study protocol for the PLAYground project

**DOI:** 10.1186/s12889-022-13991-3

**Published:** 2022-09-01

**Authors:** Allison Poulos, Pamela Hodges Kulinna

**Affiliations:** 1 College of Health Solutions, Arizona State University, Phoenix, AZ USA; 2 Mary Lou Fulton Teacher College, Arizona State University, Tempe, USA

**Keywords:** Social emotional learning, Physical activity, Improved behavior

## Abstract

**Background:**

The public health benefits of physical activity for children are well known including contributions to metabolic and cardiorespiratory health. Along with physical benefits, engaging in physical activity can support the social and emotional health of youth and promote health and well-being into adulthood. This cluster-randomized controlled trial assesses the impact of an after-school curriculum aimed at improving physically active and inclusive play to promote physical, social, and emotional health. A secondary focus is on the implementation (appropriateness, feasibility, fidelity, sustainability) of the curriculum.

**Methods:**

The PLAYground (**P**lay and **L**earning **A**ctivities for **Y**outh) project utilizes a social-ecological approach, targeting personal, behavioral, and environmental conditions, and Social Cognitive Theory (SCT) to study how a playground curriculum impacts children’s health. All elementary schools with an existing after-school program in a large, public school district in Mesa, Arizona will be eligible to participate. Seven schools will be allocated to the intervention arm in year one using random sampling stratified by school-income. In year two, the seven control schools will receive the intervention. Intervention schools will implement the research-based PlayOn!® playground curriculum to promote active and inclusive play. After-school staff will be trained to teach activities that address social and emotional skills (e.g., conflict resolution) through physical activity. Participating students will be trained as peer leaders to extend the playground activities to the recess setting. This trial will assess between-group differences in physical activity, social and emotional health indicators, and number of health and behavior incidents among students attending intervention schools and control schools. Implementation outcomes will also be assessed among program facilitators at each school site.

**Discussion:**

Enhancement of physical activity opportunities at schools has the potential for high impact and reach due to practicality. Enhancements can also improve quality pedagogy and curricula in after-school settings. Results of this project can inform practical strategies to improve existing after-school programs to prepare leaders (adults and children) to facilitate physical activity, positive social interactions, and emotional well-being.

**Trial registration:**

ClinicalTrials.gov, NCT ID NCT05470621, Registered July 22, 2022,

**Supplementary Information:**

The online version contains supplementary material available at 10.1186/s12889-022-13991-3.

## Background

The public health benefits of physical activity (PA) for children are well known, and include improved cardiorespiratory, metabolic, and mental health [1]. Despite the benefits, more than 75% of children in the United States (US) do not meet the recommended 60 minutes of moderate to vigorous physical activity (MVPA) per day [2, 3]. Inadequate PA contributes to insufficient energy expenditure, which results in weight gain [4] and obesity [5] among children. Given the tripling of obesity rates among U.S. children over the past three decades [6, 7], contributing to increased risk of cardiovascular disease [8], type 2 diabetes [9], and mental health problems such as anxiety and depression [10], incorporating regular PA into children’s lives is imperative.

Schools are crucial settings for regular PA because they provide access, structure, and systems to support healthy behaviors and health behavior change [11]. Schools are the only setting that reach nearly all children [12–15], with most children spending almost half of their waking hours at school across a period of 12 years [16]. In elementary schools, Physical Education (PE), classroom-based PA, recess, active school travel (e.g., walking/biking/rolling), and before and after school programs all contribute to MVPA accrual [17–20]. Enhancing the quality of these existing opportunities can significantly increase MVPA, and has the additional benefit of not requiring additional time for programming [21].

In the PE setting, enhancing PA typically includes the adoption of curricula and training for teachers and staff. Using either approach has been shown to increase roughly 10% of the proportion of time spent in MVPA [22]. Similar benefits have been found in the recess setting, where the most common enhancements include adding equipment, painting playground markings or designated play zones, and/or working with teachers to lead or facilitate activities. Based on a review of 13 recess interventions, the majority [12] reported statistically significant improvements in students’ PA with the percentage of time spent active during recess increasing from 5% to greater than 30% [23]. Integrating PA into classroom settings typically involves incorporating PA into lessons or adding short bursts of PA (e.g., brain boosts or brain breaks). Classroom-based PA may add an additional 2–16% increase in MVPA during activities, and an additional 2–12% increase in overall school-day MVPA [24]. Enhancing active school travel often includes coordinated programming, such as a walking school bus or bike train, which can add between 2.2–17.5 minutes of daily MVPA into children’s movement [25]. Before- and after-school activities can provide children with quality MVPA minutes, but are often more focused on academics compared to movement opportunities [26]. After-school programming is an under-utilized setting to promote PA with great reach as approximately 7.7 million children participate in after-school programs in the US [27]. In fact, a simulation study predicted that offering federally-funded after-school PA programs would have a substantial impact on reducing obesity by increasing the number of children who met daily PA recommendations by 7.7% - equating to an extra 25 minutes of daily MVPA [28].

In addition to PA, social and emotional health are critical for children. Children who have strong social and emotional foundations are better able to express their feelings in healthy ways, form positive relationships with others, express empathy, manage stress, and make responsible decisions [29]. These skills are associated with readiness for learning, engagement [30] and positive emotional well-being [29]; and predict long-term health and academic success [29, 31, 32], and emotional well-being into adulthood [33]. Improving social and emotional is crucial as depression and anxiety among children have increased over the past 20 years [34], and further exacerbated due to school closures and shifts to remote learning associated with COVID-19 pandemic [35, 36]. which constrained the ability of children to socialize with peers and receive mental health services [37]. The increase in depression symptoms and worsening of emotional well-being among children [38, 39] have prompted calls for interventions to promote positive mental health to mitigate negative effects of the pandemic [40].

This cluster-randomized trial focuses on enhancing the after-school setting by [1] integrating a curriculum to utilize playground equipment to facilitate active and inclusive play after school and [2] training students to become peer leaders to transfer skills and knowledge from the after-school setting to recess. The primary aim of this trial is to assess the effectiveness of a playground curriculum intervention to increase PA (e.g., increase moderate-to-vigorous PA (MVPA) and decrease sedentary time), improve social and emotional health, and decrease behavioral incidents among students in one large public school district in Arizona (AZ; US). We will also assess the implementation (appropriateness, feasibility, fidelity, and sustainability) of the curricular intervention to improve children’s health as a secondary outcome.

## Methods

We follow the standard reporting outlined in the SPIRT guidelines [41] with additional information recommended in the CONSORT statement for cluster randomized controlled trials [41]..

### Design and setting

This cluster randomized controlled study utilizes a parallel group, two-arm design with a 1:1 allocation ratio to measure the effectiveness of the after-school playground curriculum intervention on children’s health, with a secondary focus on assessing implementation processes to inform future efforts. For the effectiveness component (primary aim), we will randomize elementary schools in the Mesa Public School district (AZ, USA) to receive either an intervention to promote active and inclusive play through curricular enhancements or ‘usual practice’. The implementation component (secondary aim) will include a process evaluation conducted during and immediately after the intervention at program schools. All study details will be presented and discussed in consultation with district after-school and PA coordinators (who will oversee delivery of the study).

Mesa Public Schools is the largest school district in the state of Arizona, serving more than 60,000 students at 52 elementary schools. The district serves many vulnerable students as 20% of families live below the federal poverty level and only 26% of parents/guardians have earned a bachelor’s degree or higher. The school district serves a diverse student population with 50.5% white, 38.2% Hispanic, 4.6% African American, 4.3% Native American and 2.4% Asian [42]. This intervention targets children and staff at district schools that offer after-school programs (14 elementary schools).

### Participants and recruitment

#### Schools

All elementary schools (grades K-6) in the Mesa Public Schools who offer after-school programming will be eligible to participate. Schools without after-school care will be excluded. The district after-school and PA coordinator will be provided with study information via email and asked to provide written informed consent. Both coordinators will help recruit school principals to participate via email. Incentives will be offered to school principals in the form of environmental upgrades to their school playgrounds totaling approximately $50,000. Recruitment will continue until the sample of schools have consented.

#### Healthy school staff

Following school district and principal consent, a member of the research team will attend a meeting with the supervisors of participating after-school programs to provide them with a brief overview of the purpose of the study and to answer any questions. Supervisors will be asked to share recruitment materials with all after-school program staff at their school sites. The district after-school coordinator and PA coordinator will also share recruitment materials with all Physical Education (PE) teachers and recess aides at participating schools. Collectively, after-school staff, PE teachers, and recess aides will be eligible to participate as Healthy School Staff. Participating Healthy School Staff will be asked to consent to be trained to promote active and inclusive play, conduct observations of children, and participate in an interview each spring to learn about the implementation of the intervention. Participating staff will receive a $50 gift card.

#### Students

All children attending after-school programs at participating sites in grades 2–6 will be invited to take part in the study. Children attending after-school programming in grades kindergarten or first grade will be excluded due to their young age. An informational letter will be sent to parents/guardians of students at participating schools encouraging them to discuss the study procedures with their child (ren) and inviting them to participate in the study. Parental/guardian written consent will be obtained for children to participate in data collection through device-based PA assessment (accelerometry), survey, and interviews. After 1 week following the distribution of the recruitment and consent materials, Healthy School Staff will call parents/guardians who have not consented to ask if they would like to have their child (ren) participate. Student written assent will be collected among all participants. Participating students will receive a $10 gift card.

### Randomization and blinding

In the summer prior to the intervention and baseline data collection, the principal investigators of the project will use stratified randomization Gto assign schools to either the intervention or control group. Schools will be initially classified by Title 1 status - an indicator of school-level income determined by percent of students eligible for free and reduced price meals (FRPM) - according to guidelines established by the National Center for Education Statistics, where Title I schools serve more than 40% of students eligible for FRPM [43]. Then, schools within each income group will be randomly assigned using a computer-generated random number generator to either the intervention or control group to ensure even distribution. During the second year of the project, all control schools will receive the intervention. All Healthy School Staff will be aware of school group allocation due to the nature of the intervention and coordination at the school district level. Data entry staff will be blinded.

### Intervention

The intervention in this study is the implementation of the PlayOn!® playground curriculum [44] to promote active and inclusive play. PlayOn!® is a research-based curriculum that focuses on using playground structures to facilitate both free and structured play. The curriculum was developed by PlayCore with consultation from a scholar group to align with U.S. National Standards for K-12 Physical Education. Activities teach children how to initiate playground games, manage conflict, form and maintain peer relationships, develop leadership skills, and improve their physical health through increased movement.

The intervention will be delivered to Healthy School Staff at half of the schools (intervention group) in year one, and the other half of schools (control group) in year two. Students attending the after-school program at intervention schools in both year one and year two will be trained as Peer Leaders to transfer knowledge and skills from the after-school setting to recess.

#### Healthy school staff training

Healthy School Staff at the intervention schools will attend one in-person half-day training (fall 2022 for the first school group; fall 2023 for the second school group). The training will be led by the community partner, PlayCore, and supported by the research team. Healthy School Staff at each school will receive two virtual follow-up trainings (spring 2023 for the first school group; spring 2024 for the second school group).

#### Student peer leader training

Each spring (2023 for the first school group and 2024 for the second school group), students attending the after-school programs at the intervention schools will receive training to become Peer Leaders during recess.

### Control group and contamination

Components of the intervention will be delivered to the intervention schools only, and will not be provided to the control schools in year one of the study.

### Theoretical framework

This study utilizes a social-ecological approach, targeting Social Cognitive Theory (SCT) [45] to frame individual behavior change. SCT identifies personal, behavioral, and environmental conditions that impact health behavior [46]. We anticipate that the integration of a playground curriculum will improve enjoyment, knowledge, and self-efficacy (personal conditions), personal and social responsibility (behavioral conditions), and the development of peer relationships (environmental conditions) among children, leading to increased movement and decreased behavioral incidents. We frame SCT within the Theory of Expanded, Extended, and Enhanced Opportunities for youth PA promotion (TEO; 21), specifically focusing on the enhancement of existing school settings (after-school programs and recess).

### Data collection

The trial will assess between-group differences in mean minutes of light- and moderate-to-vigorous PA, SCT indicators, and number of health and behavioral incidents with data collected at baseline (Fall 2022), and approximately 6 months following the training intervention (Spring 2023). We will measure sustainability by collecting data at the first intervention school group approximately 12 months after the intervention (Fall 2023) and 18 months after the intervention (Spring 2023). Implementation outcomes (appropriateness, feasibility, fidelity, and sustainability) will be collected each spring (see Table 1).

**Table 1:** Study schedule of enrollment, interventions, and assessments

	STUDY PERIOD							
	Enrollment	Allocation	Post allocation					Close out
**TIMEPOINT**	***Pre-Trial***	**0**	***Fall 2022***	***Spring 2023***	***Summer 2023***	***Fall 2023***	***Spring 2024***	***Summer 2024***
**ENROLLMENT:**								
**Eligibility screen**	A & B							
**Informed consent**	A & B							
**Allocation**		A & B						
**INTERVENTIONS:**								
***Healthy School Staff training***			A			B		
***Student Peer Leader training***				A			B	
**PRIMARY ASSESSMENTS:**								
***Device-based physical activity***		A & B	A & B	A & B		A & B	A & B	
***Social and emotional health***								
*Healthy School Staff Observations*		A & B	A & B	A & B		A & B	A & B	
*Student surveys*		A & B	A & B	A & B		A & B	A & B	
*Student interviews*				A			B	
***Attendance and behavioral incidents***		A & B	A & B	A & B		A & B	A & B	
**SECONDARY ASSESSMENTS:**								
***Appropriateness and feasibility***				A			B	
***Fidelity***			A	A		B	B	
***Sustainability***						A	A	
***DATA CLEANING, ANALYSIS, DISSEMINATION***					A & B			A & B

A = first intervention school group

B = first control school group (year one) AND second intervention school group (year two)

### Outcomes and measures

#### Primary effectiveness outcomes

The primary outcomes in the trial will be physical activity at the student participant level as well as attendance and behavioral incidents at the school level. Intermediate indicators include SCT constructs of enjoyment, knowledge, and self-efficacy (personal conditions), personal and social responsibility (behavioral conditions), and the development of peer relationships (environmental conditions).

##### Physical activity

We will track student participants’ segmented (e.g., during recess or during after-school programming) and total PA each semester via accelerometry at both intervention and control schools using device-based data collection. Student participants will wear ActiGraph GT3X+ accelerometer devices for 24 hours over a period of 7 days (except for during water-based activities) in the fall and spring in both years. The match-box sized accelerometers (4.6 × 3.3 × 1.5 cm) are the most commonly used accelerometers in PA research [47]. Participants will wear accelerometers on their wrist to promote greater wear compliance [48]. Accelerometer data will be utilized if participants wear the devices for at least 4 days (3 weekdays and 1 weekend day) [49]. We will use two sets of cut points to determine sedentary, light-, and moderate-to-vigorous PA levels of participants in our study: one developed by Crouter et al. [50] and one by Chandler et al. [51] Because children’s activity levels change frequently, shorter epoch lengths are necessary for accurate measurement, so we will use 5 s epoch lengths recommended by both Crouter et al. and Chandler et al. Data will be segmented and processed separately to determine PA during recess, at school (including all PA opportunities such as recess, physical education, classroom PA), in after-school programming, out of school, and on weekends. These segments will be defined based on bell schedules provided by the schools, with any changes noted by Healthy School Staff.

##### Attendance and behavioral incidents

Acute bouts of PA, such as recess and out-of-school programming, have made a positive difference on classroom behavior [52]. The research team will collect de-identified attendance and behavioral referral information at the school level from the front office staff each fall and spring semester to investigate the effect of the playground intervention on school-level behavior and attendance.

##### Social cognitive theory indicators

Each fall and spring, student participants will complete a short paper questionnaire that includes measures of enjoyment [53], knowledge, and self-efficacy [54]; personal and social responsibility [55]; and peer relationships [56]. Healthy School Staff and students will also be asked to participate in focus group interviews about their experience on the playground during their afterschool program and recess each spring. The interviews will be semi-structured such that questions align with our SCT constructs and theory, but allow for flexibility and probing to understand the personal experiences of participants [56].

#### Secondary implementation outcomes

We will measure four outcomes recommended by Proctor et al. [57]: appropriateness, feasibility, fidelity, and sustainability, through surveys and interviews with Healthy School Staff and monthly observations by the research team.

##### Appropriateness

The perceived fit, relevance, and compatibility of the curriculum to improve physical, social, and emotional health and decrease behavioral incidents will be measured by survey. At follow-up in the spring at each of the first seven intervention schools in year one and second seven intervention schools in year two, Healthy School Staff will be asked to complete an online survey that includes the Intervention Appropriateness Measure (IAM), a four-item valid and reliable scale [58].

##### Feasibility

The extent to which the PlayOn!® curriculum can be successfully used within after-school settings will be measured by survey. At follow-up in the spring at each of the first seven intervention schools in year one and second seven intervention schools in year two, Healthy School Staff will be asked to complete an online survey that includes the Feasibility of Intervention Measure (FIM), a four-item valid and reliable scale [58].

##### Fidelity

The degree to which the intervention was implemented as intended and described in the protocol, or fidelity, is a critical component of the evaluation of health behavior interventions [59]. We will measure fidelity using data from monthly observations completed by the research team at each of the seven intervention schools in year one and each of the seven intervention schools in year two. The observations will be conducted using a tool developed by the research team that aligns with the five dimensions of intervention fidelity identified by Proctor et al. [57]: adherence, exposure/dose, quality of delivery, component differentiation, and participant involvement.

##### Sustainability

Because the majority of enhancements to programs are effective in increasing PA during the intervention period [60], the sustained engagement or integration of the curriculum within the after-school and recess setting will be measured approximately 12- and 18- months following the completion of the intervention using data from observations completed by the research team using the fidelity tool.

### Data management

Data will be stored securely and accessible only to primary researchers and statisticians. Confidential participant data will be stored securely and not linked to survey responses. No data monitoring committee is needed as this study involves minimal risk.

### Data analysis

Intervention effects on the primary trial outcomes (at each follow-up time point) will be assessed using general linear mixed effects models to account for the clustering of individual measurements within schools. These will include fixed effects for treatment group (intervention vs control), time (baseline and follow-up), and interaction term (intervention x time); include school as a random effect; and will be adjusted for covariates related to the outcome (eg, student sex, age). Analyses related to physical, social, and emotional health will be performed with the child (nested within a school) as the unit of analysis. Analysis related to attendance and behavioral change will be performed with the school as the level of analysis. Separate analyses will be performed at each follow-up time point. Schools will be included in analyses if they meet at least 80% adherence to the protocol.

Secondary implementation outcomes will be assessed using descriptive statistics for Healthy Staff and Student survey data (appropriateness and feasibility) and data from monthly observations (fidelity and sustainability). Frequencies, means, and standard deviations will be calculated to describe the distributions, measures of central tendency, and dispersion of item responses. Data from Healthy School Staff and Student interviews will be analyzed using a matrix analysis to summarize and organize data in alignment with Proctor’s dimensions (adherence, exposure/dose, quality of delivery, component differentiation, and participant involvement), and content analysis to identify salient themes for each school, by school-level income, and collectively among intervention school groups. We will utilize a deductive approach, but allow for additional themes to emerge.

### Sample size calculation

The average enrollment of schools in the study district is 610 students [61]. The average enrollment in all 14 after school programs across schools in the study district is 420 students. Using an estimate of a 70% participation rate among after-school programs and assuming 20% loss-to-follow-up, a sample of 7 intervention and 7 control schools will provide a sample of approximately 294 students (147 intervention and 147 control) at follow-up. Assuming 80% power and an alpha of 0.05, the sample will be sufficient to detect an effect size ranging from .279–.360 (intra class correlation coefficient of 0.01–0.05 respectively), similar to other school-based PA interventions reporting small overall effects [62, 63].

### Research trial governance

This study will utilize an advisory group consisting of researchers, school staff, and community practitioners to oversee all aspects of the planning, implementation, and evaluation of the project. The group will develop and implement all components of the trial according to study protocol. Dissemination of results will include oversight from all members of the group. Journal publications and reports will follow author guidelines outlined by the International Committee of Medical Journal Editors (ICMJE). Auditing to monitor adherence to study interventions will be conducted by research staff during Healthy School Staff observations.

### Trial discontinuation or modification

While we do not anticipate any events to occur that would warrant discontinuing the trial, the intervention may be modified or discontinued at the request of participants. Any adverse events will be reported to the Arizona State University Ethics Committee (primary approval committee). Modifications to the protocol will be updated in the trial registration record and any changes will be reported in study publications.

## Discussion

Incorporating opportunities for movement into the comprehensive school day is a public health strategy to improve children’s physical, social, and emotional health. Enhancing existing opportunities, such as recess and after-school programming, is a particularly promising approach to achieve impact and reach because of its practicality [21]. Integrating movement into the culture of schools can have a sustained population-level impact.

This protocol outlines the design and methods that will be used to assess the impact of a curricular intervention to facilitate active and inclusive play on the playground during after-school programming and recess to improve students’ physical, social, and emotional health. Given the limited evidence and opportunity to focus on after-school programming, this trial will provide practical and theoretically-informed methods to guide PA interventions in school-based settings.

## Supplementary Information

**Additional file 1.**

## Data Availability

Not applicable.

## Abbreviations

PA: Physical activity
US: United States
MVPA: Moderate-to-vigorous physical activity
PE: Physical education
AZ: Arizona
